# The value of procalcitonin and urinary NGAL in the prediction of acute pyelonephritis and kidney scarring in pediatric patients with a history of febrile urinary tract infection: a systematic review and meta-analysis

**DOI:** 10.1007/s00467-025-06885-0

**Published:** 2025-07-31

**Authors:** Nikolaos Gkiourtzis, Anastasia Stoimeni, Panagiota Michou, Nikolaos Charitakis, Konstantinos Cheirakis, Vasileios Liakos, Kali Makedou, Nikoleta Printza, Charalampos Antachopoulos, Despoina Tramma

**Affiliations:** 1 4th Department of Pediatrics, School of Medicine, Department of Health Sciences, “G. Papageorgiou” General Hospital, Aristotle University of Thessaloniki, 54124 & Ring Road Municipality of Pavlou Mela Area N. Evkarpia, 56403 Thessaloniki, Greece; 2 Pediatric Department, G. Gennimatas General Hospital, Thessaloniki, Greece; 3 1St Department of Nephrology, School of Medicine, Department of Health Sciences, Hippokration General Hospital, Aristotle University of Thessaloniki, Thessaloniki, Greece; 4 3rd Department of Pediatrics, School of Medicine, Department of Health Sciences, Ippokrateio General Hospital, Aristotle University of Thessaloniki, Thessaloniki, Greece; 5 Laboratory of Biochemistry, School of Medicine, Department of Health Sciences, AHEPA University Hospital, Aristotle University of Thessaloniki, Thessaloniki, Greece; 6 1 st Department of Pediatrics, School of Medicine, Department of Health Sciences, Ippokrateio General Hospital, Aristotle University of Thessaloniki, Thessaloniki, Greece

**Keywords:** Acute pyelonephritis, Vesicoureteral reflux, Kidney scarring, Biomarkers, Meta-analysis

## Abstract

**Background:**

Children with febrile urinary tract infections (fUTI), especially those with acute pyelonephritis (APN) and vesicoureteral reflux (VUR), may face several complications, especially kidney scarring. Different biomarkers, such as urinary neutrophil gelatinase-associated lipocalin (uNGAL) and procalcitonin (PCT), have been studied for predicting kidney scarring without the need for a DMSA scan.

**Objectives:**

This systematic review and meta-analysis examines the role of biomarkers in pediatric patients with a history of fUTI and their prognostic value in the diagnosis of APN and prediction of kidney scarring.

**Data sources:**

A search through major databases (MEDLINE/PubMed and Scopus) was conducted from inception until January 2, 2025. The mean difference (95% CI) was applied. A *p* < 0.05 was considered statistically significant.

**Results:**

The systematic review included 2300 participants from 28 studies. Procalcitonin and uNGAL were higher in individuals with scarring after a fUTI episode compared to those without scarring (*p* = 0.035 and *p* < 0.0001, respectively). In addition, PCT was higher in patients with APN compared to those with lower UTI (*p* < 0.0001). Finally, the good and moderate overall diagnostic accuracy (I^2^ = 29.8%) of PCT in predicting APN (AUC: 0.861) and uNGAL (I^2^ = 16.8%) in predicting kidney scarring after an episode of fUTI (AUC: 0.74), respectively, should be considered.

**Conclusions:**

This study showed that PCT and uNGAL can detect APN and kidney scarring after fUTI. More studies should be conducted exploring the role of other biomarkers in scarring after an episode of fUTI in pediatric patients with or without VUR.

**Graphical abstract:**

**Figure:**
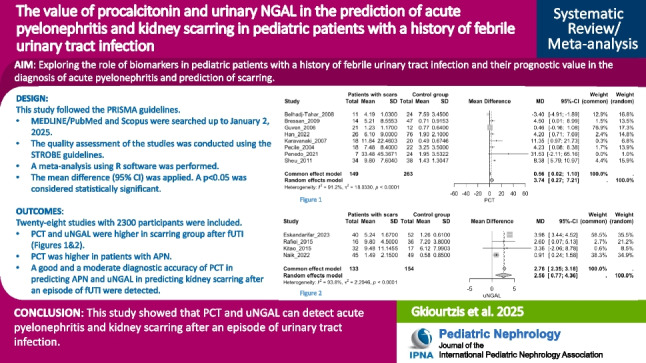
A higher resolution version of the Graphical abstract is available as Supplementary information

**Supplementary Information:**

The online version contains supplementary material available at 10.1007/s00467-025-06885-0.

## Introduction

Urinary tract infection (UTI) is one of the most common bacterial infections in children, representing the most common cause of bacterial infection in infants and toddlers [1–3]. Children with febrile urinary tract infections (fUTI) experience symptoms of acute illness and face potential complications, such as kidney scarring [4, 5]. Approximately one-third of patients with recurrent fUTI will develop kidney scarring [5]. Recurrent episodes of UTI (rUTI) are more common in children with a history of vesicoureteral reflux (VUR) compared to children without VUR [6, 7]. A recent post hoc analysis of two multicenter studies showed that the risk of developing kidney scarring after one episode of fUTI is 2.8%, after two episodes 25.7%, and after three episodes 28.6% [8].

Acute pyelonephritis (APN) is a leading cause of permanent kidney damage, and each new episode increases the risk of kidney scarring by 10 to 30% [1, 8–12]. A large cohort study, including 1221 children with a first episode of symptomatic UTI, revealed that boys more commonly have dysplastic or hypoplastic kidneys with or without VUR, and girls have scars related to rUTI episodes [13, 14].

Technetium-99 m dimercaptosuccinic acid (DMSA) scan is the gold standard of kidney scarring detection according to international guidelines [15, 16]. NICE guidelines recommend performing a DMSA scan 4–6 months after episodes of atypical UTI in children under three years of age and rUTI in children of any age [17].

Neutrophil gelatinase-associated lipocalin (NGAL), kidney injury molecule (KIM-1), liver fatty acid binding protein (L-FABP), pentraxin 3 (PTX3), and procalcitonin (PCT) have been studied during the last 25 years in an effort to develop a model for predicting and detecting kidney scarring without the need for DMSA scan [5, 18–25]. To date, only two systematic reviews have been conducted on the prognostic role of PCT in kidney scarring in pediatric patients with a history of APN [19, 26].

Therefore, we conducted a systematic review and meta-analysis examining the role of biomarkers in pediatric patients with a history of fUTI and their prognostic value in the diagnosis of APN and predicting kidney scarring.

## Materials and methods

### Study registration and search strategy

This study followed Preferred Reporting Items for Systematic Reviews and Meta-Analyses (PRISMA) guidelines [27]. A protocol has been registered in the OSF platform (https://osf.io/ayq43/). We searched MEDLINE/PubMed and Scopus up to January 2, 2025, using a basic search strategy developed for MEDLINE/PubMed and modified for other research engines using the terms: “pyelonephritis” OR “UTI” OR “vesicoureteral reflux” OR “VUR” AND “neutrophil gelatinase associated lipocalin” OR “NGAL” OR “kidney injury molecule-1” OR “KIM-1” OR “liver fatty acid binding protein” OR “L-FABP” OR “pentraxin 3” OR “PTX3” OR “procalcitonin” AND “kidney scarring” OR “renal scar”. References from included studies were checked for additional eligible studies. Clinicaltrials.gov, PROSPERO, OSF, and grey literature were also reviewed to identify relevant studies and trials, ensuring no duplication. No limitations for publication year were applied. Only publications written in English language were considered.

### Eligibility criteria

The research question was defined according to the criteria established in a prespecified protocol [28]. Our primary intention was to include clinical trials, specifically randomized controlled trials and/or observational studies, that examined the association of biomarkers of kidney scarring in pediatric patients with fUTI or VUR. No restrictions were applied to the definition of UTI. Individuals with other congenital urinary lesions, except VUR, were excluded. No restrictions for urine sampling methods and diagnosis of UTI were applied. Systematic reviews, case reports, case series, and studies with adults were excluded.

### Study procedure (collection and extraction of data)

Two independent reviewers (NG and PM) conducted the literature search, imported all records into a reference management tool (rayan.qcri.org), and removed duplicates [29]. Initially, the remaining studies were assessed by title and abstract, followed by full-text review. Any discrepancies were resolved through discussion with a third author (DT). Two reviewers (AS and NC) independently extracted data from eligible studies using a pre-specified form. Disagreements were resolved with the help of a third reviewer (NG). If any study characteristics were missing, we contacted the corresponding authors for additional information.

### Quality assessment of the included studies

The quality assessment of the studies was conducted using the Strengthening the Reporting of Observational Studies in Epidemiology (STROBE) guidelines. This includes 22 checklist items about the study quality of the title and abstract, introduction, methodology, results, discussion, conclusions, and funding [30]. Three scoring options for each item are available: 0 if not fulfilled, 1 if fulfilled, and NA if not applicable [31]. A STROBE adherence score of over 85% is graded as “excellent”, a score of 70% to 84% as “good”, a score of 50% to 69% as “fair”, and below 50% as “poor”. Two reviewers (AS and NC) assessed the quality of each study, with disagreements resolved through consensus with the help of a third reviewer (NG). Finally, funnel plots were used for publication bias assessment [32].

### Outcome measurements

The primary outcomes were defined as the difference in biomarker levels between study groups, such as serum and urinary NGAL (sNGAL and uNGAL), serum and urinary KIM-1 (sKIM-1 and uKIM-1), serum and urine L-FABP (sL-FABP and uL-FABP), serum and urine PTX3 (sPTX3 and uPTX3), and PCT. In addition, the difference in biomarker-to-creatinine ratios between the study groups was the secondary outcome. Only biomarkers with sufficient data were included in the systematic review and meta-analyzed. After the inclusion of systematic review studies, we examined the accuracy of biomarkers in predicting an APN episode and kidney scarring.

### Statistical analysis and data synthesis

A meta-analysis employing R software (Version 4.3.2) was performed, using the “meta” package [33]. The group differences were estimated using mean difference (MD) for continuous outcomes with 95% confidence intervals (CI). Forest plots were used to illustrate the weighted outcomes with the 95% CI. Small study effects, including publication bias, were evaluated using funnel plots and Egger’s test when enough studies were available [34]. Heterogeneity between the studies was assessed using the I^2^ test, where I^2^ value < 40% was set as low, 30–60% as moderate, 50–90% as substantial and 75–100% as considerable [32]. A sensitivity analysis was conducted in cases of considerable heterogeneity, followed by further exploration using a Baujat plot [35]. A meta‐analysis of the accuracy, using the “mada” package, was conducted [36]. Summary sensitivities and specificities are clinically interpretable only when the studies included in a meta-analysis employ a standard cut-off value. Therefore, we estimated sensitivity at points on the Summary Receiver Operator Curves (SROC) that corresponded to the median specificity observed in the studies included in the meta-analysis [37]. The sensitivity and specificity results were presented in figures for each study. A *p* < 0.05 was considered statistically significant.

### Terminology

Considering the inconsistency in different UTI categories and the fact that no restrictions were applied to the definition of UTI during study design, we should add clarifications on terminology. A fUTI is an episode of UTI accompanied by fever, and APN is the parenchymal inflammation secondary to bacterial invasion of a kidney [1]. The two terms are often used interchangeably. Recurrent UTI is defined as two or more UTI episodes within six months or three or more UTI episodes within a year [38]. Finally, a lower UTI is a UTI episode of the lower urinary tract (cystitis or urethritis).

## Results

**Fig. 1:**
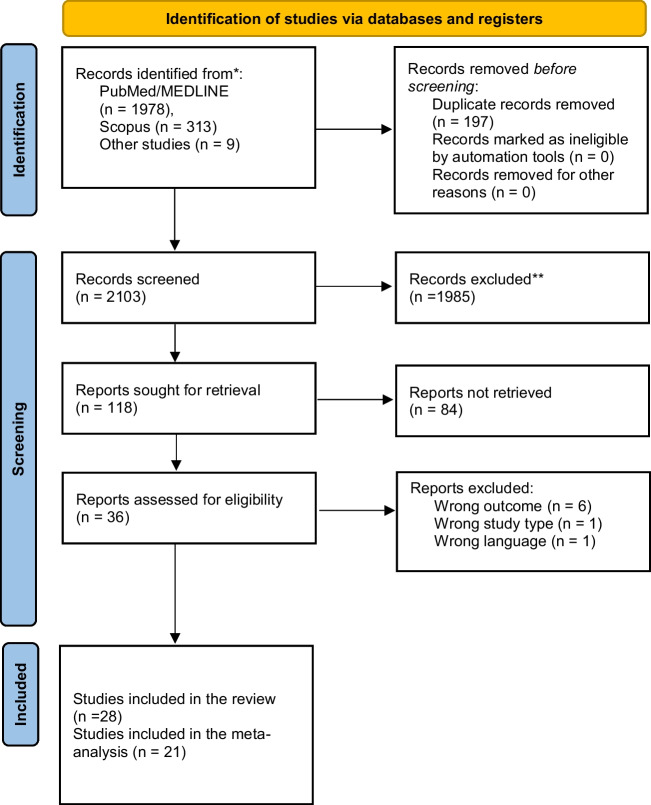
The PRISMA 2020 flow chart

The initial search identified 2300 articles (Supplementary Table 1). After duplicate detection, 1985 records were removed (Fig. 1). After title and abstract screening, we assessed 36 full-text studies for eligibility (﻿Supplementary Table 2). Finally, 28 studies, with 2300 individuals, published between 1998 and 2023, were included in the systematic review [21–25, 39–61].

### Study characteristics

The baseline characteristics of the included studies are presented in Table 1. The mean age of the study participants was between 3.5 months (1.8) to 10.6 years (2.9). The main background of nine studies’ patients was the relation of VUR with kidney scarring [21–23, 25, 39, 42, 44, 45, 53] and in 20 studies the relation of UTI [49] and fUTI [43, 45, 46, 48, 50–52, 54, 55, 57–61] or APN [24, 40, 41, 47, 56], with or without VUR, with kidney scarring. In total, 811 individuals with VUR as the main background [21–23, 25, 39, 42, 44, 45, 53] and 1581 individuals with UTI (UTI, fUTI, and APN) [24, 40, 41, 43, 45–52, 54–61] were included in the systematic review. Over half of the eligible studies evaluated the predictive role of PCT on kidney scarring [40, 41, 43, 46–49, 51, 54–59, 61]. Three of the included studies were cross-sectional [39, 44, 53], 21 studies were prospective [21, 22, 24, 40–43, 45–47, 49–51, 54–61], and four were retrospective studies [23, 25, 48, 52]. Eleven studies were conducted in Europe [40, 41, 43, 44, 49, 51, 54–56, 59, 61], and 17 studies were conducted in Asia [21–25, 39, 42, 45–48, 50, 52, 53, 57, 58, 60]. Finally, 21 studies were included in the meta-analysis [23, 24, 40, 41, 43, 45–51, 53–61]. Meta-analyses that included two or fewer studies are not presented.

**Table 1:** Baseline characteristics of included studies

Study ID	Registration number	Country	Type of study	Background	Study groups		Mean age	Male (%)	Patients included in the study (ITT number)	Fever Duration	Clinical question	Type of biomarker	Biomarker assessment
Amiri 2022	IR. UMSHA.REC.1399.143	Iran	Cross-sectional	VUR	Mild VUR	Unilateral VUR	59.1 months (34.7)	20.6	63	N/A	Kidney scarring	pNGAL, uNGAL, uNGAL/Cr	Acute phase
					Moderate VUR	Bilateral VUR							
					Severe VUR								
					Lower UTI								
Becerir 2019	Pamukkale University Ethics Committee (IRB number 60116787–020/549639)	Turkey	Prospective	VUR	VUR with kidney scarring		6.0 years	35.2	88	N/A	Kidney scarring	sPTX3, uPTX3, uPTX3/Cr	Acute phase
					Kidney scarring								
					VUR								
					Control								
Belhadj-Tahar 2008	N/A	France	Prospective	Episode of APN	Fibrosis		27.2 months (5.54)	24	183	N/A	Kidney scarring	PCT	Acute phase
					Absence		39.9 months (6.8)						
Benador 1998	N/A	France	Prospective	fUTI episode with or without VUR	Lower UTI		36.0 months (9.0)	28.3	80	N/A	Kidney scarring	PCT	Acute phase
					APN		42 months (8.0)						
Benzer 2017	N/A	Turkey	Prospective	VUR	VUR with kidney scarring		6.5 years (3.4)	31.2	107	N/A	Kidney scarring	uL-FABP, uL-FABP/Cr	Follow-up
					VUR								
					Control		6.1 years (2.7)	47.2					
Bressan 2009	N/A	Italy	Prospective	First episode of APN with or without VUR	APN with kidney scarring		3.9 months (4.8)	50	61	N/A	Kidney scarring	PCT	Acute phase
					APN without kidney scarring		4 months (4.9)	51.1					
Colceriu 2023	Ethics Committee of the Emergency Clinical Hospital for Children in Cluj-Napoca, Romania (no.168/28.05.2021)	Romania	Cross-sectional	VUR	VUR		6.9 years (12.3)	53.8	78	N/A	Kidney scarring	uNGAL, uNGAL/Cr, uNGAL/IL-37	Simultaneously
					Control		7.6 years (13.0)						
Eskandarifan 2023	Kurdistan University of Medical Sciences (IR.MUK.REC.1395/165)	Iran	Prospective	VUR and episode of APN	VUR, APN with kidney scarring		22.7 months (14.0)	35.8	92	N/A	Kidney scarring	uNGAL	Follow-up
					VUR, APN without kidney scarring								
Gurgoze 2005	N/A	Turkey	Prospective	UTI episode with or without VUR	APN		39.6 months (33.8)	24.6	114	N/A	Kidney scarring	PCT	Acute phase
					Lower UTI								
Guven 2006	N/A	Turkey	Prospective	Episode of APN	Normal DMSA scan		4.42 years	6.0	33	N/A	Kidney scarring	PCT	Acute phase
					Positive DMSA scan								
Han 2022	CHA University Institutional Review Board approved this study and the consent procedure (No. CHA 2021–09-043)	Korea	Retrospective	Recurrent fUTI episode	Kidney scarring		4.2 months (2.1)	92.3	134	N/A	Kidney scarring	PCT	Acute phase
					Control		3.5 months (1.8)	72.4					
Ichino 2010	N/A	Japan	Retrospective	VUR	VUR		49.9 months	64.5	62	Ν/Α	Kidney scarring	uNGAL	Simultaneously
					Control		50.5 months						
Karavanaki 2007	N/A	Greece	Prospective	UTI episode with or without VUR	fUTI and abnormal DMSA scan		3.4 years (7.1)	34.5	58	N/A	Kidney scarring	PCT	Acute phase
					fUTI and normal DMSA scan								
					Afebrile UTI								
Kitao 2015	Hospital ethics committee approval (No. H140732)	Japan	Prospective	fUTI episode with or without VUR	fUTI with kidney scarring		6.4 years (11.2)	71.4	49	N/A	Kidney scarring	uNGAL/Cr, L-FABP/Cr	Follow-up
					fUTI without kidney scarring		3.1 years (2.9)						
Kotoula 2009	N/A	Greece	Prospective	First UTI episode with or without VUR	UTI with cortical defect		40.7 months (80.6)	24.6	57	N/A	Kidney scarring	PCT	Acute phase
					UTI without cortical defect								
Lee 2020	Korea University Ansan Hospital, IRB No. 2018AS0140	Korea	Retrospective	First fUTI episode with or without VUR	fUTI with kidney scarring		20.5 months (31.6)	71.4	78	N/A	Kidney scarring	pNGAL	Acute phase and follow-up
					fUTI without kidney scarring		12.8 months (22.8)	48.0					
Naik 2022	N/A	India	Cross-sectional	VUR	VUR with kidney scarring		36.0 months (36.8)	79.8	94	N/A	Kidney scarring	uNGAL, uNGAL/Cr, uKIM-1, uKIM-1/Cr	Acute phase
					VUR without kidney scarring		13.7 months (15.3)						
Orive 2012	N/A	Spain	Prospective	First fUTI episode	fUTI with kidney scarring		N/A	36.0	140	N/A	Kidney scarring	PCT	Acute phase
					fUTI without kidney scarring								
Parmaksız 2016	Başkent University Institutional Review Board and Ethics Committee (Project no: KA11/153)	Turkey	Prospective	VUR	VUR with kidney scarring		8.3 years (2.6)	30.1	153	N/A	Kidney scarring	uNGAL/Cr, uKIM-1/Cr, uL-FABP/Cr	Simultaneously
					VUR without kidney scarring		9.2 years (3.2)						
					Resolved VUR with kidney scarring		9.7 years (3.2)						
					Resolved VUR without kidney scarring		10.6 years (2.9)						
					Control		9.5 years (2.9)						
Pecile 2004	N/A	Italy	Prospective	First fUTI episode with or without VUR	fUTI with kidney scarring		19 months	31	100	N/A	Kidney scarring	PCT	Acute phase
					fUTI without kidney scarring								
Penedo 2021	N/A	Spain	Prospective	Episode of APN	APN with kidney scarring		43.3 months (55.5)	14.3	31	48 h (44.1)	Kidney scarring	PCT	Acute phase and follow-up
					APN without kidney scarring		7.3 months (6.9)	45.8		56 h (56.7)			
Prat 2003	N/A	Spain	Prospective	First fUTI episode with or without VUR	fUTI with kidney scarring		N/A	N/A	77	N/A	Kidney scarring	PCT	Acute phase
					fUTI without kidney scarring								
Rafiei 2015	N/A	Iran	Prospective	Episode of APN with or without VUR	APN with kidney scarring		44.1 months (25.1)	3.7	54	N/A	Kidney scarring	uNGAL, uNGAL/Cr	Acute phase
					APN without kidney scarring		34.8 months (37.2)						
Sheu 2011	N/A	Taiwan	Prospective	First fUTI episode with or without VUR	APN		5.0 months	58.9	76	N/A	Kidney scarring	PCT	Acute phase
					Lower UTI								
Smolkin 2002	N/A	Israel	Prospective	fUTI episode with or without VUR	APN		18.3 months (26.5)	28.3	64	N/A	Kidney scarring	PCT	Acute phase
					Lower UTI		16.7 months (26.1)						
Toker 2013	N/A	Turkey	Retrospective	VUR	VUR		6.6 years (2.4)	39.4	74	N/A	Kidney scarring	uKIM-1, uKIM-1/Cr	Simultaneously
					Control		6.4 years (4.1)						
Tuerlinchx 2005	N/A	Belgium	Prospective	First fUTI episode	Normal DMSA scan		44.0 months	22.2	63	N/A	Kidney scarring	PCT	Acute phase
					Initial abnormal DMSA scan								
Yamanouchi 2018	Kansai Medical University Ethics Committee (No. 1518)	Japan	Prospective	fUTI episode with or without VUR	fUTI with kidney scarring		5.0 years (9.2)	73.9	37	4 days (5.5)	Kidney scarring	uNGAL, uL-FABP	Follow-up
					fUTI without kidney scarring		4.1 years (8.1)	57.1		3.3 days (4.1)			

APN: acute pyelonephritis; Cr: creatinine; DMSA scan: Technetium-99 m dimercaptosuccinic acid scan; fUTI: febrile urinary tract infection; ID: identification; ITT: intention to treat; (p/u) KIM-1: (plasma/urine) Kidney Injury Molecule-1; protein-1; (u) L-FABP: (urine) liver fatty acid binding protein; N/A: Not Applicable; (p/u) NGAL: (plasma/urine) neutrophil gelatinase-associated lipocalin; PCR: Protein to Creatinine Ratio; PCT: procalcitonin; (s/u) PTX3: (serum/urine) pentraxin 3; SD: standard difference; UK: United Kingdom; USA: United States of America; UTI: urinary tract infection; VUR: vesicoureteral reflux.

### Quality assessment of the included studies

﻿Supplementary Table 3 presents the quality assessment of the 28 included studies [21–25, 39–61], following STROBE guidelines [30, 31]. Eleven studies had a “good” quality grade [22, 39, 42, 43, 45, 47, 51–53, 56, 60], 14 studies had a “fair” quality grade [21, 23–25, 40, 41, 44, 46, 48–50, 55, 57, 61], and only three studies had a “poor” quality grade [54, 58, 59].

### Primary outcome

#### Procalcitonin

##### **Procalcitonin and kidney scarring**

**Fig. 2:**
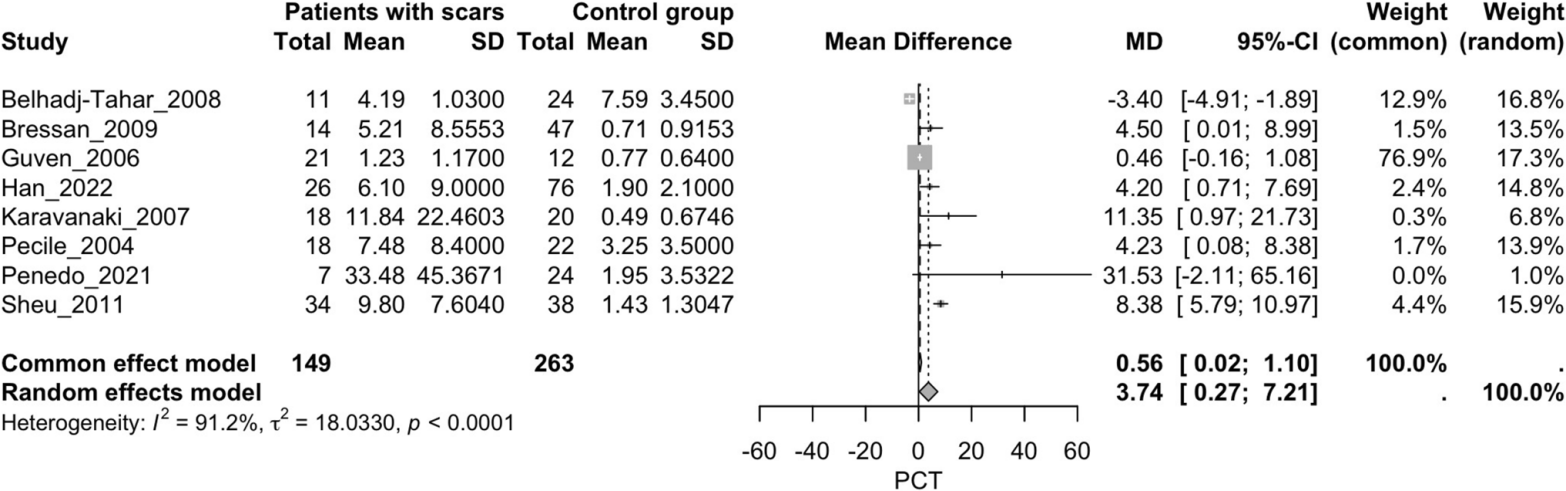
Forest plot assessing the association of procalcitonin levels and kidney scarring between the study groups

Overall, eight studies assessed this outcome, including patients with kidney scars after a fUTI episode and a control group without scars [40, 43, 47–49, 55–57]. During the episode of fUTI, the first group exhibited significantly higher PCT values compared to the second group (MD = 3.74, 95% CI [0.27, 7.21], *p* = 0.035, I^2^ = 91.2%) (Fig. 2). A Baujat plot was conducted to explore sources of heterogeneity, which identified the study by Sheu et al. as having a high influence on the overall result (﻿Supplementary Fig. 1) [57]. Sensitivity analysis excluding this study did not substantially reduce the heterogeneity (Supplementary Figs. 2 and 3).

#### Urinary NGAL

##### **Urinary NGAL and kidney scarring**

**Fig. 3:**
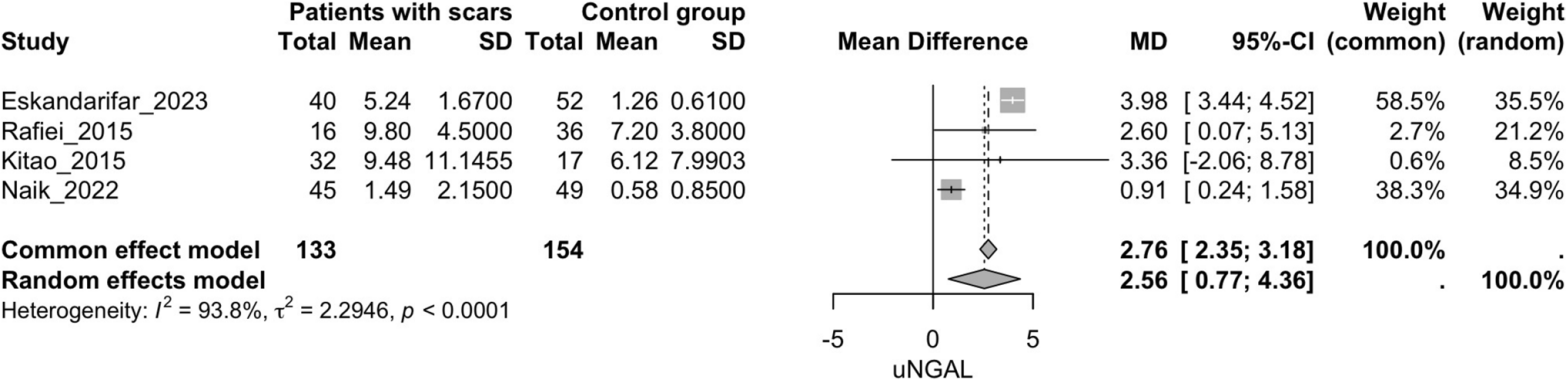
Forest plot assessing the association of uNGAL and kidney scarring between the study groups; uNGAL: urinary neutrophil gelatinase-associated lipocalin

**Fig. 4:**
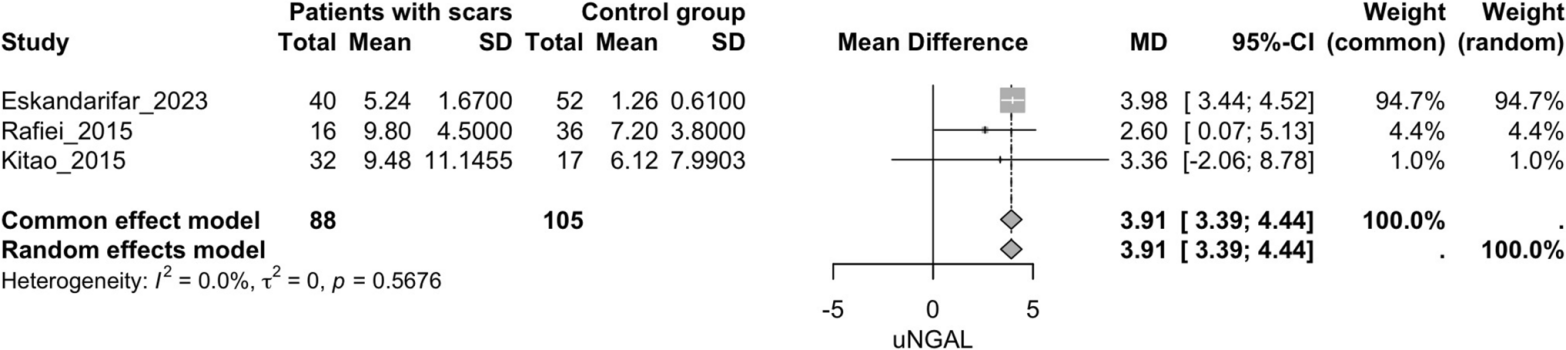
Forest plot assessing the association of uNGAL and kidney scarring between the study groups after sensitivity analysis; uNGAL: urinary neutrophil gelatinase-associated lipocalin

Four studies evaluated this outcome [24, 45, 50, 53]. The group with kidney scarring exhibited significantly higher uNGAL values compared to the group without scars (MD = 2.56, 95% CI [0.76, 4.36], *p* = 0.005, I^2^ = 93.8%) (Fig. 3). After sensitivity analysis, omitting Naik et al. [53], the group with kidney scars still exhibited significantly higher uNGAL values compared to the control group (MD = 3.91, 95% CI [3.39, 4.344], *p* < 0.0001, I^2^ = 0%) (Fig. 4).

## Secondary outcomes

### Procalcitonin

**Fig. 5:**
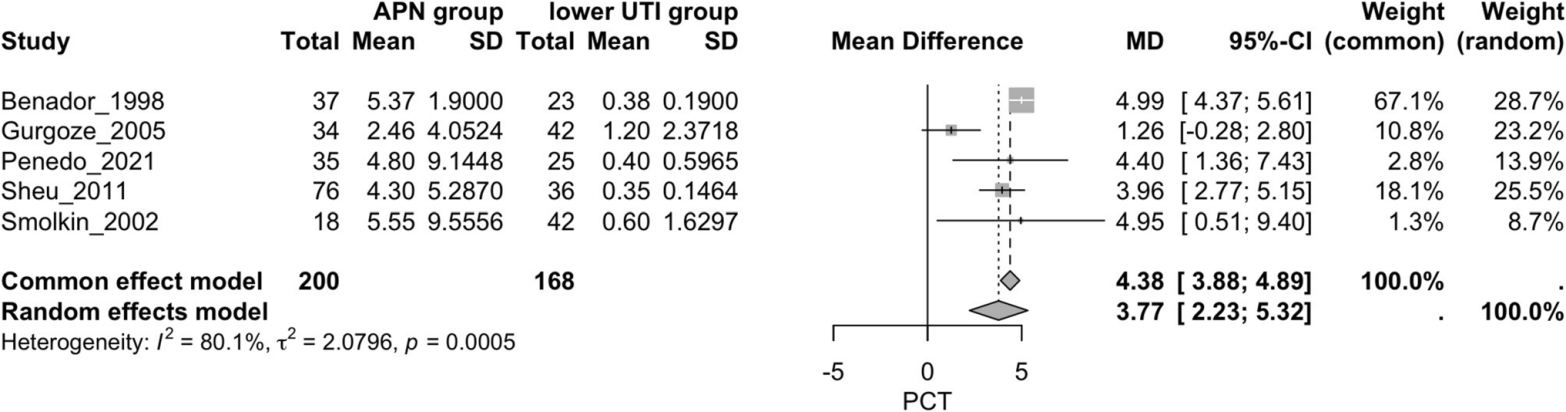
Forest plot assessing the association of procalcitonin levels and APN between the study groups; APN: acute pyelonephritis

**Fig. 6:**
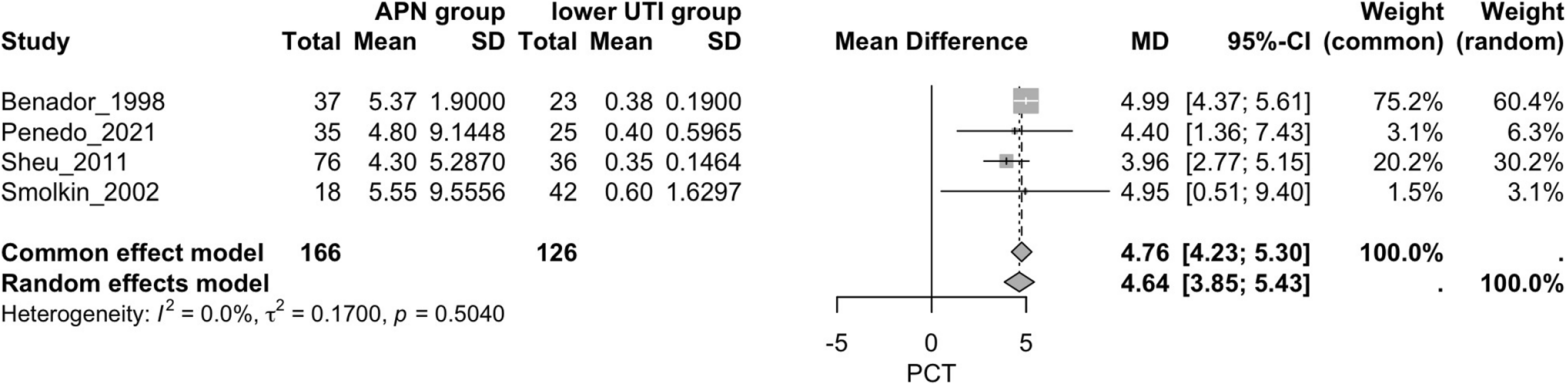
Forest plot assessing the association of procalcitonin levels and APN between the study groups after sensitivity analysis; APN: acute pyelonephritis

A. *Procalcitonin and APN*
  Overall, five studies assessed this outcome, including patients with APN and patients with lower UTI [41, 46, 56–58]. The APN group exhibited significantly higher PCT values compared to the lower UTI group (MD = 3.77, 95% CI [2.23, 5.32], *p* < 0.0001); however, there was substantial heterogeneity (I^2^ = 80.1%) (Fig. 5). Following a sensitivity analysis (Supplementary Fig. 4), the study by Gurgoze et al. was omitted [46]. After this adjustment, the APN group still showed significantly higher PCT values compared to the lower UTI group (MD = 4.64, 95% CI [3.85, 5.43], *p* < 0.001), with reduced heterogeneity (I^2^ = 0%) (Fig. 6).
B. *Accuracy of PCT in predicting APN*
  A bivariate random-effects meta-analysis was conducted to evaluate the diagnostic performance of PCT in predicting APN. The pooled sensitivity was estimated at 0.84 (95% CI [0.67, 0.93]), indicating a high ability of the test to correctly identify true positives. The corresponding pooled false positive rate was 0.24 (95% CI [0.14, 0.37]), translating to a specificity of approximately 0.76 (95% CI [0.63, 0.86]). A strong negative correlation (*r* = − 0.76) was observed between sensitivity and false positive rate. The area under the ROC curve (AUC) was 0.861, indicating good overall diagnostic accuracy (I^2^ = 29.8%) (Supplementary Figs. 5 and 6).

### Urinary NGAL

**Fig. 7:**
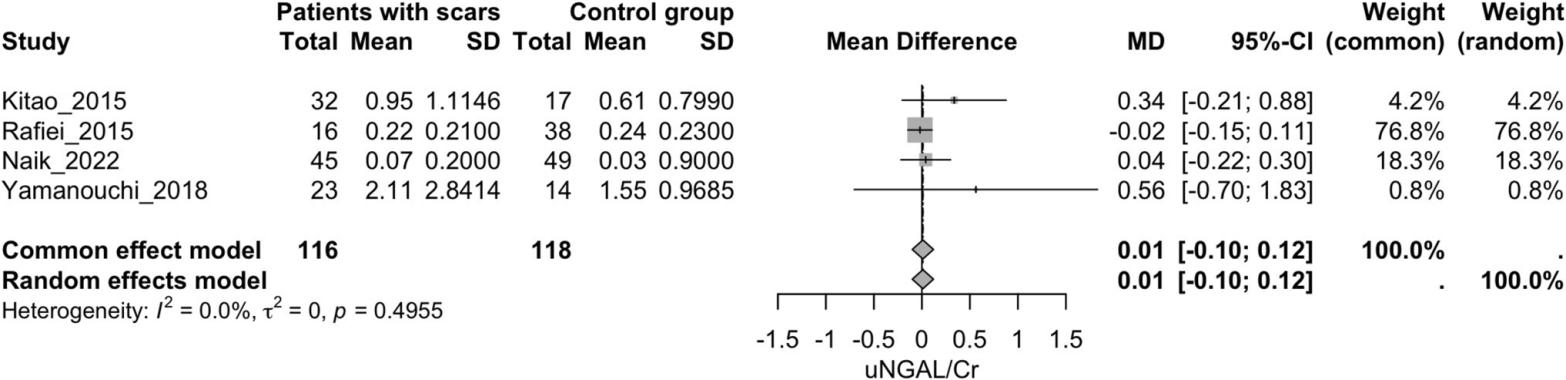
Forest plot assessing the association of uNGAL to creatinine ratio and kidney scarring; uNGAL: urinary neutrophil gelatinase-associated lipocalin

A. *Urinary NGAL to creatinine ratio (uNGAL/Cr) and kidney scarring*
  Four studies evaluated this outcome [24, 50, 53, 60]. The patient group did not exhibit higher uNGAL/Cr values compared to the control group (MD = 0.01, 95% CI [−0.1, 0.12], *p* = 0.8, I^2^ = 0%) (Fig. 7).
B. *Accuracy of urinary NGAL in predicting kidney scars*
  A hierarchical SROC (HSROC) model was fitted using restricted maximum likelihood estimation to evaluate the diagnostic accuracy of the test. The pooled sensitivity was estimated at 0.73 (95% CI: 0.66–0.80), and the pooled false positive rate was 0.27 (95% CI [0.19, 0.37]). The area under the ROC curve (AUC) was 0.74, indicating moderate discriminatory ability of the index test. Additionally, the partial AUC, restricted to the range of observed false positive rates and normalized, was 0.71. The model showed low heterogeneity (I^2^ = 16.8%), suggesting low between-study variability in diagnostic accuracy among the included studies (Supplementary Figs. 7 and 8).

## Discussion

Acquired kidney scarring is more common after episodes of rUTI and high-grade VUR [6, 7]. Early recognition of possible risk factors of kidney scarring is crucial to initiate adequate treatment and continue with follow-up. Shaikh et al. have concluded that temperature ≥ 39 °C, any pathogen other than *Escherichia coli*, abnormal ultrasonographic findings, polymorphonuclear cell count ≥ 60%, C-reactive protein (CRP) ≥ 40 mg/L, and the presence of VUR ≥ third grade were associated with the development of kidney scarring in pediatric patients with a first UTI [9]. The predictive ability of the combination of elevated temperature, abnormal ultrasonographic findings, and non-*Escherichia coli* pathogens was only 3–5% less than the predictive ability of models that depended on voiding cystourethrogram or blood draw. The patients who align with the above characteristics represent a particularly high-risk group, of whom 30% will eventually develop scarring. Almost half of the patients with eventual scarring could be identified via this model (temperature, ultrasonographic findings, pathogen). Nevertheless, scarring in many patients may remain undetected. Blood or urine biomarkers could add value in kidney scarring detection after a fUTI episode. Until now, no systematic review or meta-analysis has examined the association of different biomarkers, except PCT, in kidney scarring in children with a history of VUR or after an episode of fUTI. Two systematic reviews have studied the relationship between PCT in children with APN [19] and fUTI [26] and subsequent kidney scarring. Our initial goal was to conduct an extensive systematic review of all the available clinical studies examining the association of biomarkers in pediatric patients with a history of VUR or UTI and kidney scarring.

Urine NGAL, also known as lipocalin-2, is a small 25-kD protein sensitive to tubular cell damage [62]. It is produced as a product in the distal nephron in response to Gram negative bacterial infections and kidney injury, being an excellent marker of kidney disease activity and progression [63, 64]. In addition, PCT is a 116-amino acid propeptide of calcitonin and an early marker of bacterial infection, rising in response to bacterial endotoxins with high sensitivity and specificity [19, 65, 66]. According to previous studies, PCT levels over 0.5 ng/ml may predict early kidney parenchymal involvement and kidney scarring after an episode of UTI [19, 26]. Specifically, Leroy et al. showed that PCT levels over 0.5 ng/ml had 71% sensitivity and 72% specificity for APN detection and 88% sensitivity and 50% specificity for kidney scarring detection [19]. In addition, the studies included in Mantadakis et al.’s meta-analysis showed that PCT had a sensitivity of 59–100% and specificity of 23–90% with a serum PCT cut-off value of 0.5–0.6 ng/ml for the detection of acute kidney injury [26].

Previous reviews, systematic reviews, and meta-analyses have explored and presented mainly the role of PCT during APN or in kidney scarring [18, 19, 26, 67]. Leory & Gervaix collected all the available studies until 2011, showing that PCT has a good diagnostic accuracy for APN, with a specificity ranging from 70 to 100% and a sensitivity ranging from 70 to 97% [67]. Mantadakis et al.’s meta-analysis, using a cut-off value of 0.5 to 0.6 ng/ml, demonstrated a pooled diagnostic odds ratio of PCT for culture and DMSA-proven UTI with kidney parenchymal involvement of 14.25 (95% CI [4.7, 42.2]) [26]. Thus, this level may predict the presence of kidney injury, distinguishing APN from lower UTI. Mantadakis et al. revealed that their results are feasible mainly for febrile children with an episode of fUTI. In 2013, Leroy et al. conducted a meta-analysis exploring the role of PCT in the prediction of APN and kidney scarring in children with UTI. A total of 1011 children were included. Procalcitonin showed a significantly higher area under the ROC curve than CRP or white blood cell count for APN and kidney scarring. A cut-off value of 0.5 ng/ml showed a specificity of 72% and a sensitivity of 71% for APN and 50% specificity and 79% sensitivity for kidney scarring. Finally, Kosmeri et al. conducted a review of risk factors and predictors of kidney scarring after a fUTI episode in children [18]. Apart from PCT, the authors presented the role of cystatin C, angiotensinogen, tissue inhibitor of metalloproteinase-1, matrix metalloproteinase-9, interleukin-8, and NGAL for predicting kidney scarring, failing to give a clear answer, possibly due to the lack of an adequate number of included studies. According to the data presented, only endothelin-1 showed great sensitivity and specificity in the detection of kidney scarring after an episode of UTI [68].

Our systematic review included 2300 individuals from 28 studies (811 individuals with VUR as the main background and 1581 individuals with UTI and subsequent scarring), examining the association of biomarkers with APN and fUTI with subsequent kidney scarring. We have tried to address the long-term concerns regarding the most severe complication of UTI and how we can accurately predict it. A broad search through the main databases and screening of all the available reviews of our systematic search was conducted, trying to eliminate the risk of any study being missed. No other study has been conducted trying to explore the significance of the available biomarkers in the prediction of kidney scarring after an episode of fUTI or their accuracy in APN detection.

According to our meta-analysis, PCT was higher during APN episodes compared to lower UTI episodes, while it showed good diagnostic accuracy for APN episodes. Both PCT and uNGAL were higher in patients with kidney scarring after an episode of UTI compared to individuals without scars. Meanwhile, the uNGAL-to-creatinine ratio was not different between individuals with and without kidney scarring. Unfortunately, only Rafiei et al. and Naik et al. presented the urinary creatinine levels of the two study groups [24, 53]. According to these studies, the group of patients with kidney scarring had significantly higher urinary creatinine levels compared to the groups without scarring. This finding is based on the fact that acute kidney injury may lead to increased risk of chronic kidney disease due to tubulointerstitial damage and fibrosis [69, 70]. Finally, HSROC revealed a moderate discriminatory ability of the accuracy of uNGAL in predicting kidney scarring after an episode of UTI. Additionally, the partial AUC showed low heterogeneity, suggesting low between-study variability in diagnostic accuracy of the included studies.

Nevertheless, our study has limitations that should be acknowledged. Unfortunately, the exploration of the role of different biomarkers in the relationship between VUR and kidney scarring could not be conducted due to the lack of adequate studies for meta-analysis. We were able to conduct meta-analyses only for PCT and uNGAL, as there was not enough data for other biomarkers. Although a great number of participants were included in the study, the number of participants in each meta-analysis was limited. Although statistical significance was reached regarding uNGAL and kidney scarring, only four studies were included in the analysis [24, 45, 50, 53]. Therefore, further confirmation is needed to establish its importance in clinical practice. Additionally, patients with or without VUR could not be separated in each study group. The definition of UTI in different studies was inconsistent (differences in threshold cfu/ml of positive urine culture, urine sampling method, and APN diagnosis with physical examination and urine culture or DMSA scan). Additionally, the studies included in our study used different inclusion criteria regarding the episodes of UTI (first episode of APN or fUTI, any episode of APN or fUTI, and rUTI). Therefore, as Mantadakis et al. revealed previously for PCT, the results of this meta-analysis apply mainly to febrile children with an episode of fUTI [26]. Furthermore, the “eternal” problem of inability to differentiate patients with congenital lesions and those with acquired scarring following APN may increase the bias of our results. Finally, a wide range of ages was noted among the study participants. Infants and young toddlers are more likely to have an APN compared to older children and teenagers, who usually have episodes of lower UTI [16]. This observation could influence the accuracy of biomarkers in the diagnosis of APN or kidney scarring after an episode of fUTI.

It is significant to mention that the prediction of APN and scarring in pediatric patients with a history of fUTI using PCT and uNGAL may guide or even alter the role of imaging studies during the acute phase and follow-up period, as well as the short- and long-term management of these patients. In addition, the effect of acquired scarring on kidney function should be explored. Grade 1 kidney scarring seems to be the most common outcome in children with VUR and fUTI episodes [6], but there is not adequate literature to explore the true impact of such lesions on long-term kidney function. Furthermore, there have been limited reports regarding the risk factors of nephropathy in children with VUR [71]. Male gender, severe VUR, history of rUTI, and older age at diagnosis may be the most dominant risk factors [8, 71, 72]. In addition, congenital anomalies of the kidney and urinary tract (CAKUT) may have a significant impact on the development of scarring or nephropathy [5]. In contrast, the *PREDICT* study suggested that CAKUT did not increase the risk of more severe kidney damage in 2 years, even after APN [73]. Therefore, this is another topic that needs to be explored.

## Conclusions

This systematic review and meta-analysis explores all available studies examining the association of biomarkers in pediatric patients with a history of fUTI and kidney scarring. This study revealed the significant role of PCT and uNGAL in accurately detecting APN and predicting kidney scarring after an episode of fUTI. Since no meta-analyses could be conducted for other biomarkers or patients with VUR, due to the lack of available data, more clinical studies should be conducted exploring the role of different biomarkers in kidney scarring after an episode of fUTI or in pediatric patients with a history of VUR.

## Supplementary information

Below is the link to the electronic supplementary material.

Graphical abstract (PPTX 346 KB)

ESM 2 (PNG 72.9 KB)

ESM 3 (PNG 175 KB)

ESM 4 (PNG 219 KB)

ESM 5 (PNG 139 KB)

ESM 6 (PNG 69.5 KB)

ESM 7 (PNG 78.5 KB)

ESM 8 (PNG 78.0 KB)

ESM 9 (PNG 72.5 KB)

ESM 10 (DOCX 15.5 KB)

ESM 11 (DOCX 17.6 KB)

ESM 12 (DOCX 22.5 KB)

## Data Availability

Data is available online.
