# Supplementary material for: The value of procalcitonin and urinary NGAL in the prediction of acute pyelonephritis and kidney scarring in pediatric patients with a history of febrile urinary tract infection: a systematic review and meta-analysis
Source: Pediatr Nephrol. 2025 Jul 31;41(2):323–37. doi: 10.1007/s00467-025-06885-0 (PMC12727856; doi:10.1007/s00467-025-06885-0)
Supplement: Supplementary file 1 — Graphical abstract (PPTX 346 KB) [file 467_2025_6885_MOESM1_ESM.pptx]

## Slide 1
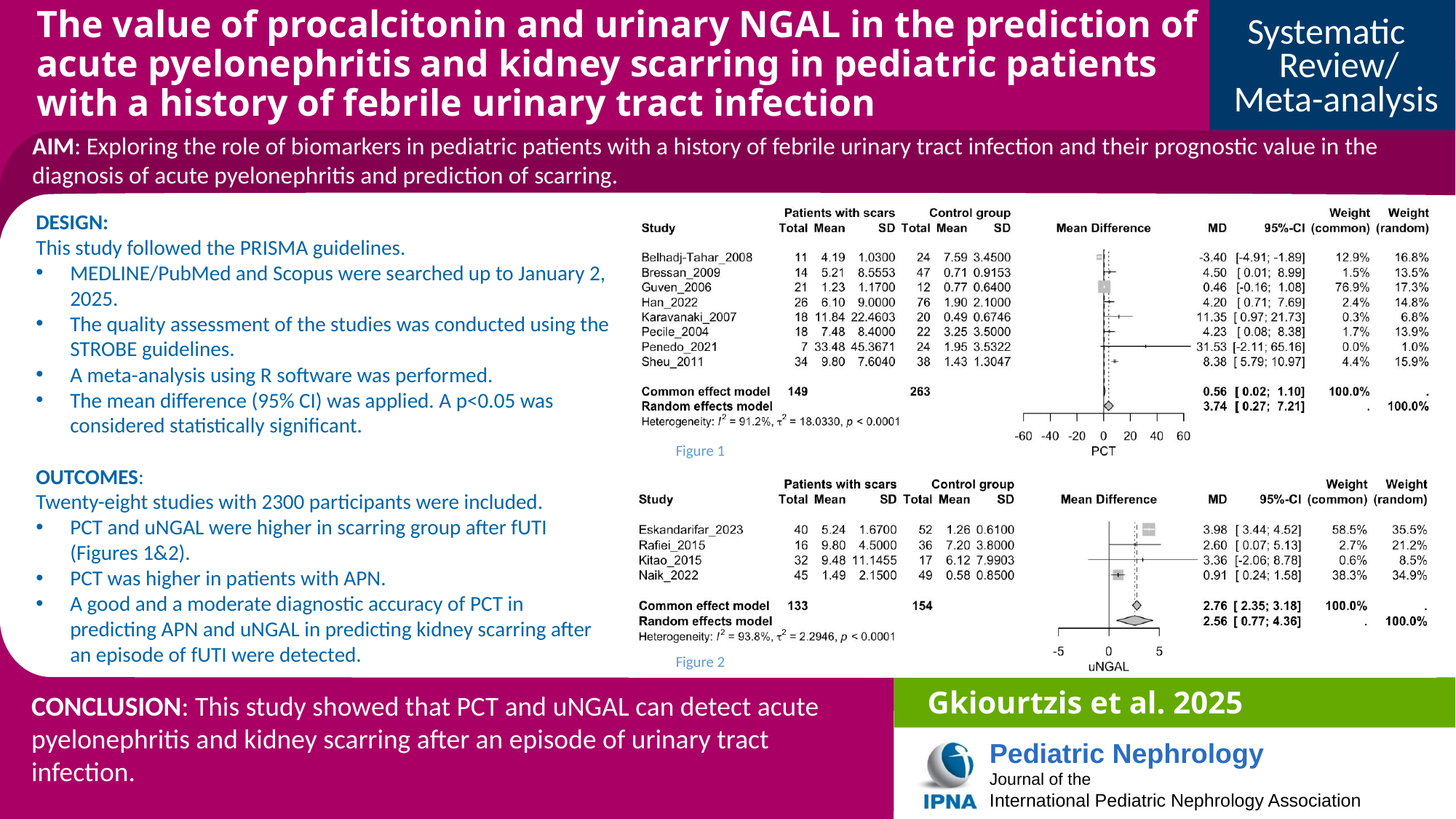

The value of procalcitonin and urinary NGAL in the prediction of acute pyelonephritis and kidney scarring in pediatric patients with a history of febrile urinary tract infection
AIM: Exploring the role of biomarkers in pediatric patients with a history of febrile urinary tract infection and their prognostic value in the diagnosis of acute pyelonephritis and prediction of scarring.
DESIGN:
This study followed the PRISMA guidelines.
MEDLINE/PubMed and Scopus were searched up to January 2, 2025.
The quality assessment of the studies was conducted using the STROBE guidelines.
A meta-analysis using R software was performed.
The mean difference (95% CI) was applied. A p<0.05 was considered statistically significant.
OUTCOMES:
Twenty-eight studies with 2300 participants were included.
PCT and uNGAL were higher in scarring group after fUTI (Figures 1&2).
PCT was higher in patients with APN.
A good and a moderate diagnostic accuracy of PCT in predicting APN and uNGAL in predicting kidney scarring after an episode of fUTI were detected.
Figure 1
Figure 2
Gkiourtzis et al. 2025
CONCLUSION: This study showed that PCT and uNGAL can detect acute pyelonephritis and kidney scarring after an episode of urinary tract infection.
