# Supplementary figures and images for: The value of procalcitonin and urinary NGAL in the prediction of acute pyelonephritis and kidney scarring in pediatric patients with a history of febrile urinary tract infection: a systematic review and meta-analysis
Source: Pediatr Nephrol. 2025 Jul 31;41(2):323–37. doi: 10.1007/s00467-025-06885-0 (PMC12727856; doi:10.1007/s00467-025-06885-0)

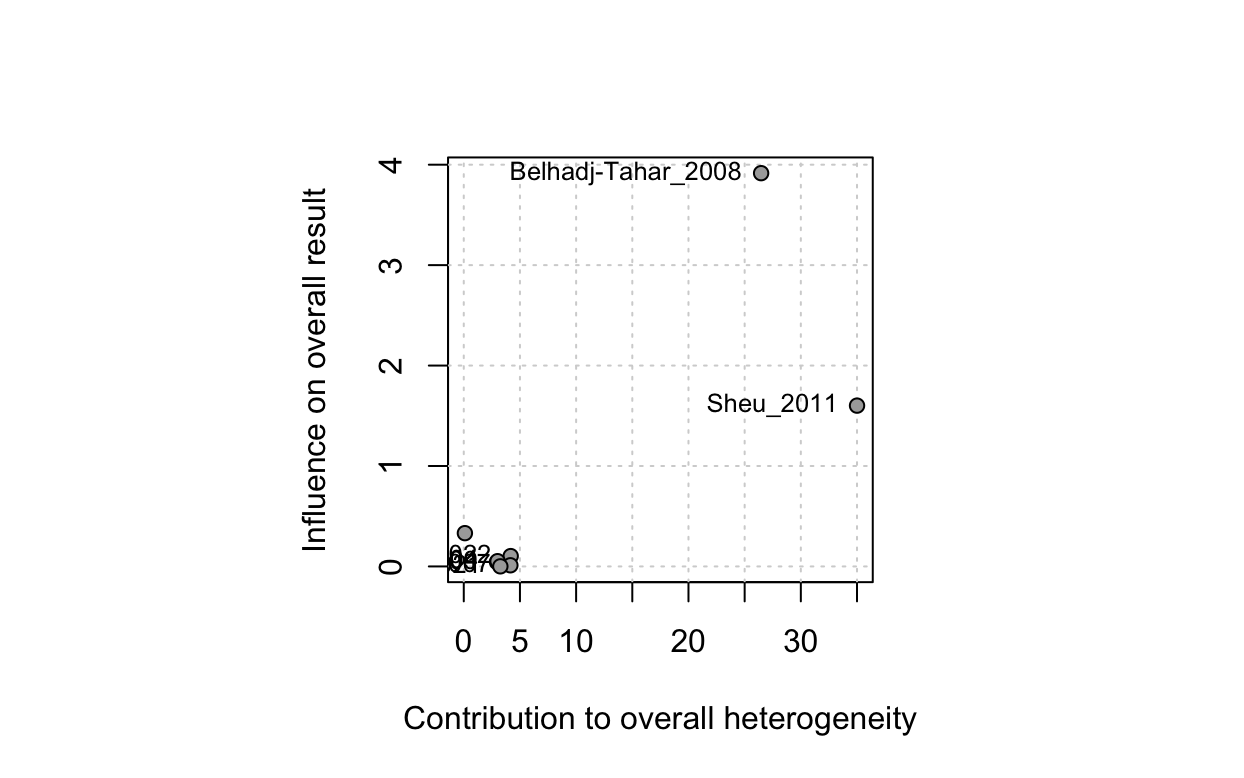

Supplement: Supplementary file 2 — ESM 2 (PNG 72.9 KB) [file 467_2025_6885_MOESM2_ESM.png]

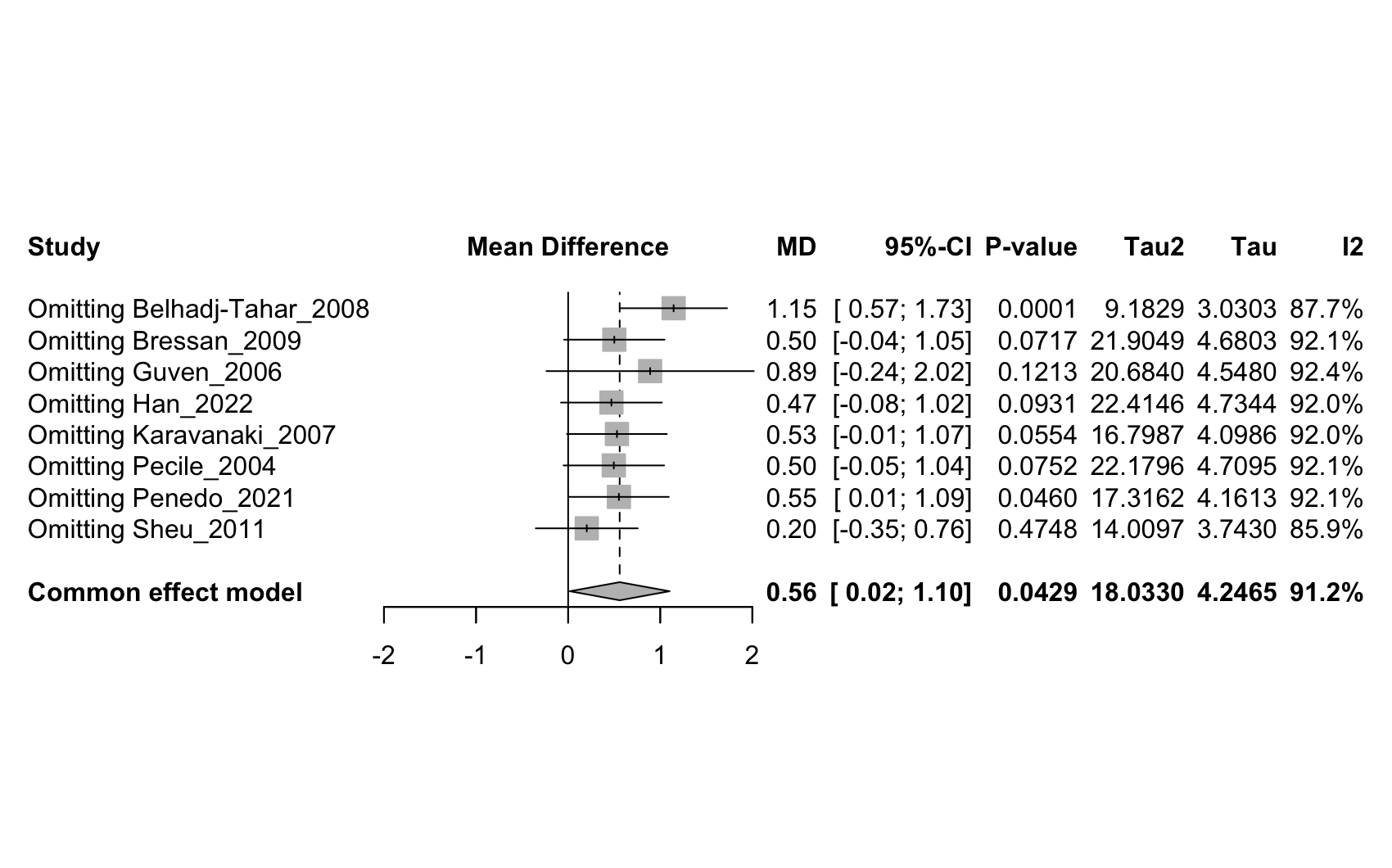

Supplement: Supplementary file 3 — ESM 3 (PNG 175 KB) [file 467_2025_6885_MOESM3_ESM.png]

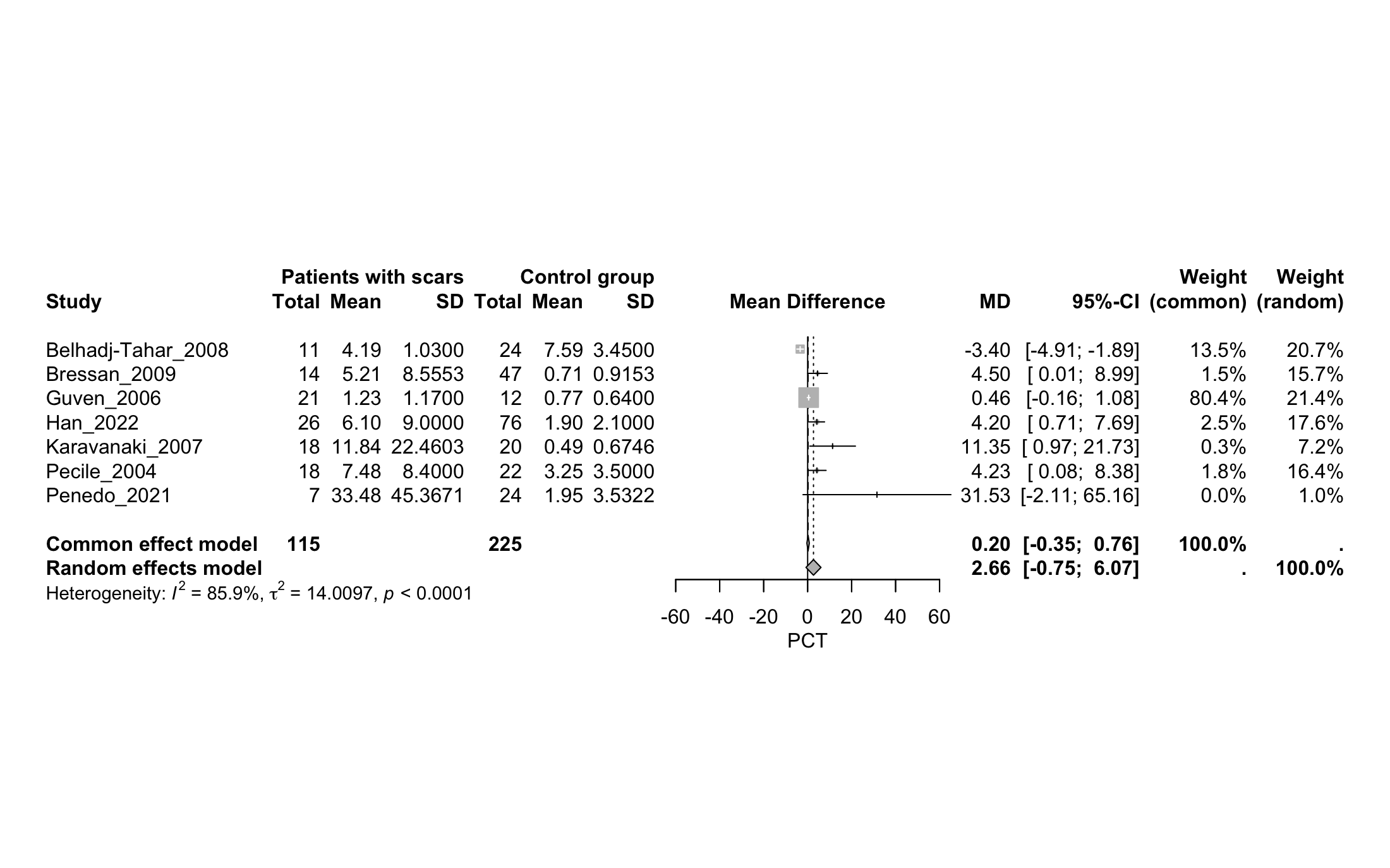

Supplement: Supplementary file 4 — ESM 4 (PNG 219 KB) [file 467_2025_6885_MOESM4_ESM.png]

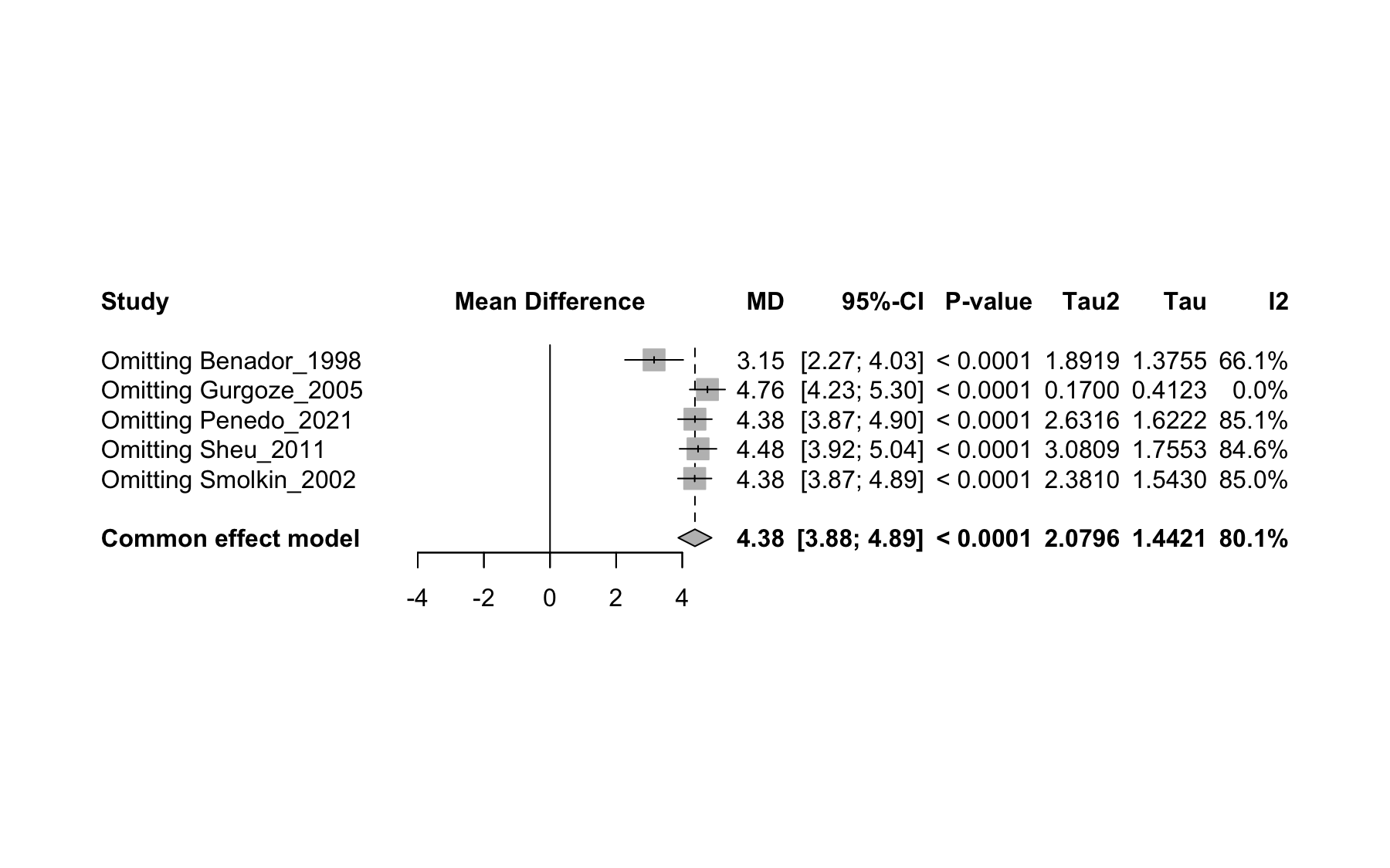

Supplement: Supplementary file 5 — ESM 5 (PNG 139 KB) [file 467_2025_6885_MOESM5_ESM.png]

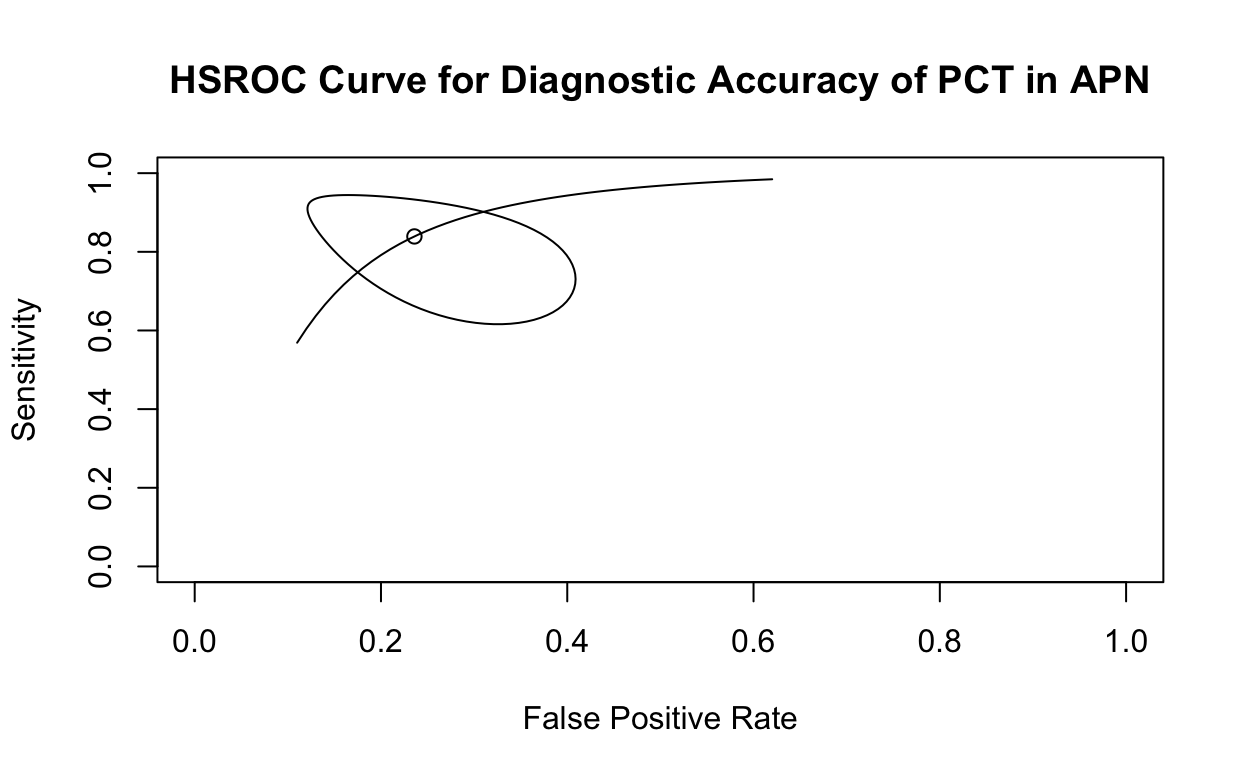

Supplement: Supplementary file 6 — ESM 6 (PNG 69.5 KB) [file 467_2025_6885_MOESM6_ESM.png]

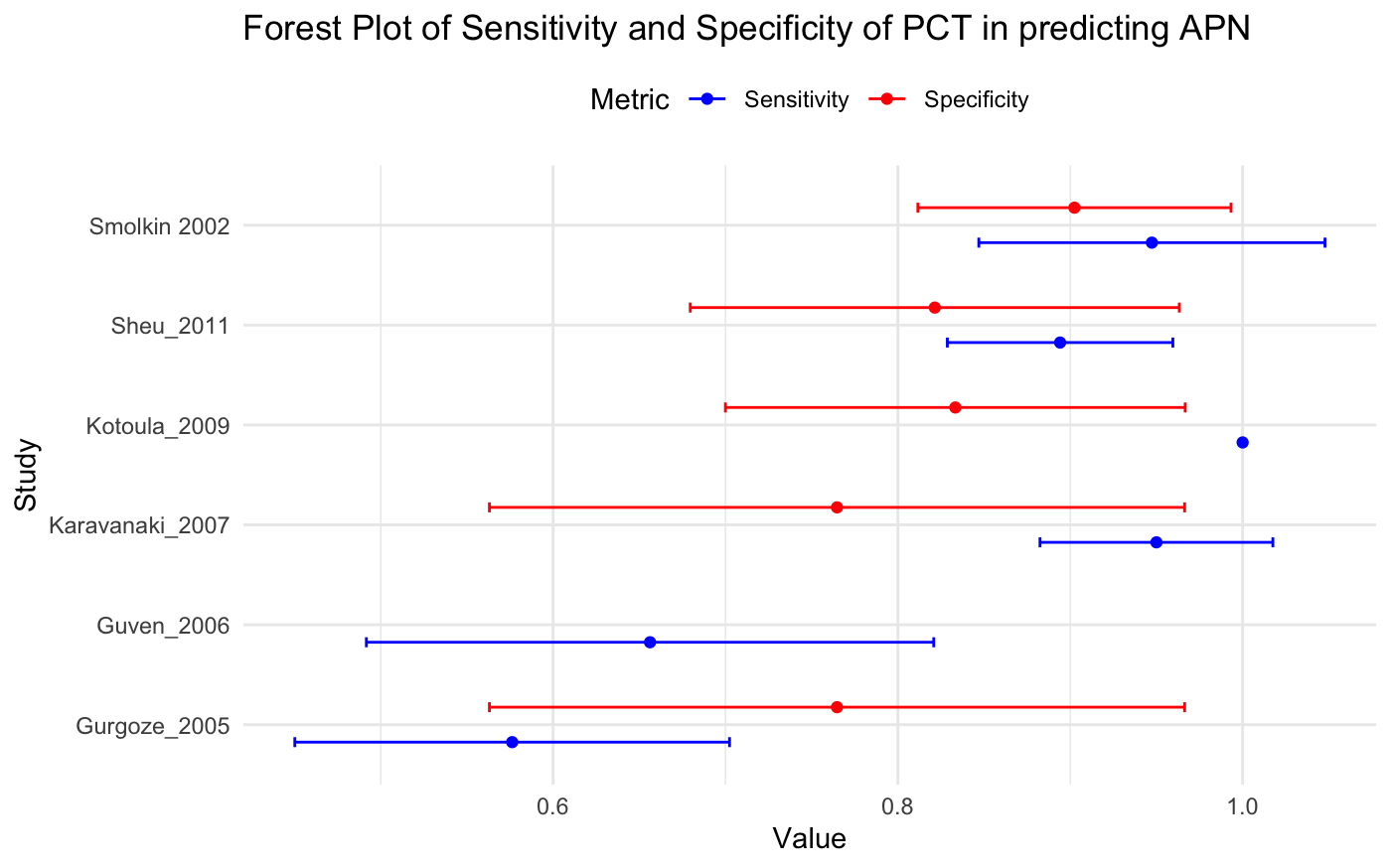

Supplement: Supplementary file 7 — ESM 7 (PNG 78.5 KB) [file 467_2025_6885_MOESM7_ESM.png]

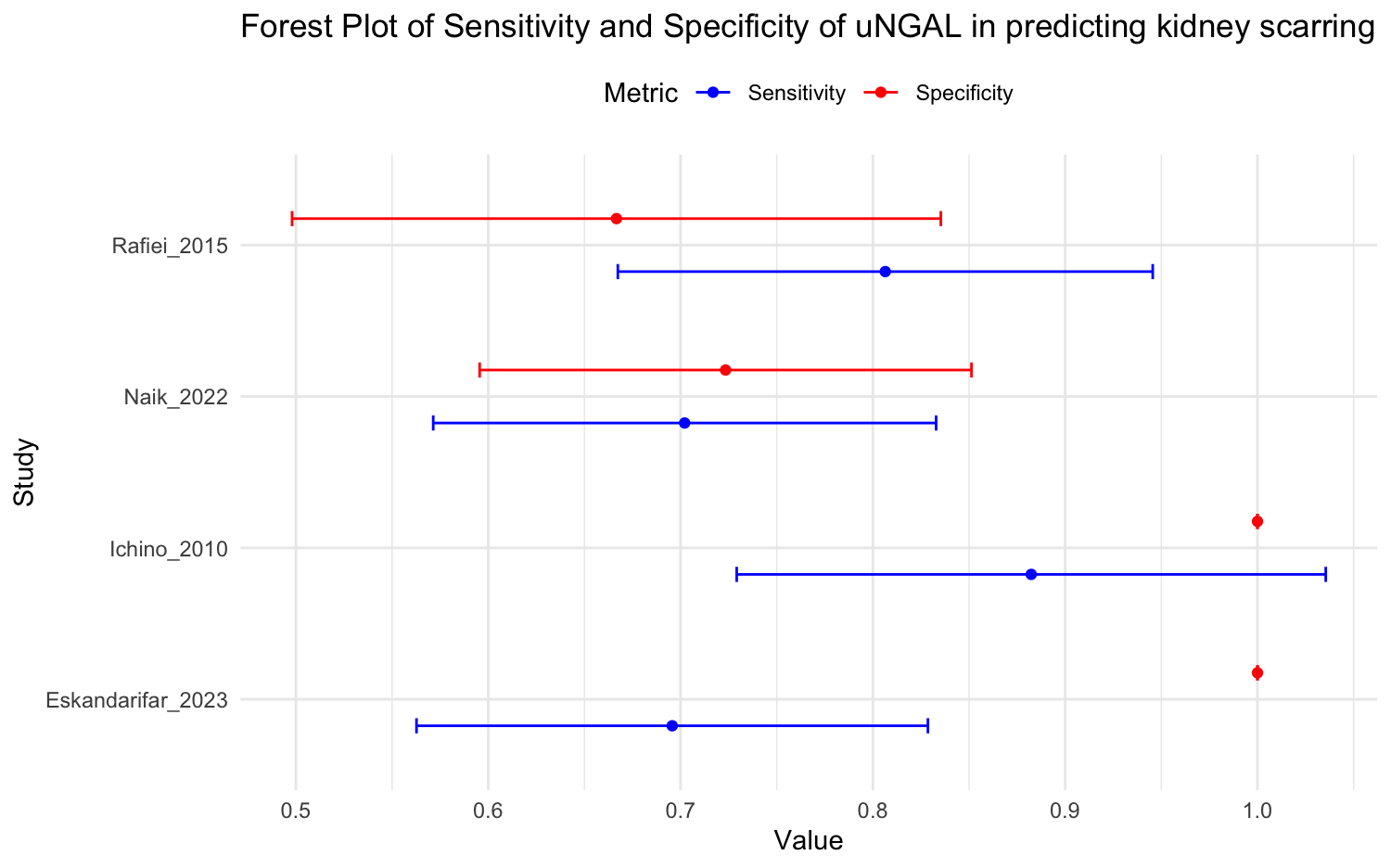

Supplement: Supplementary file 8 — ESM 8 (PNG 78.0 KB) [file 467_2025_6885_MOESM8_ESM.png]

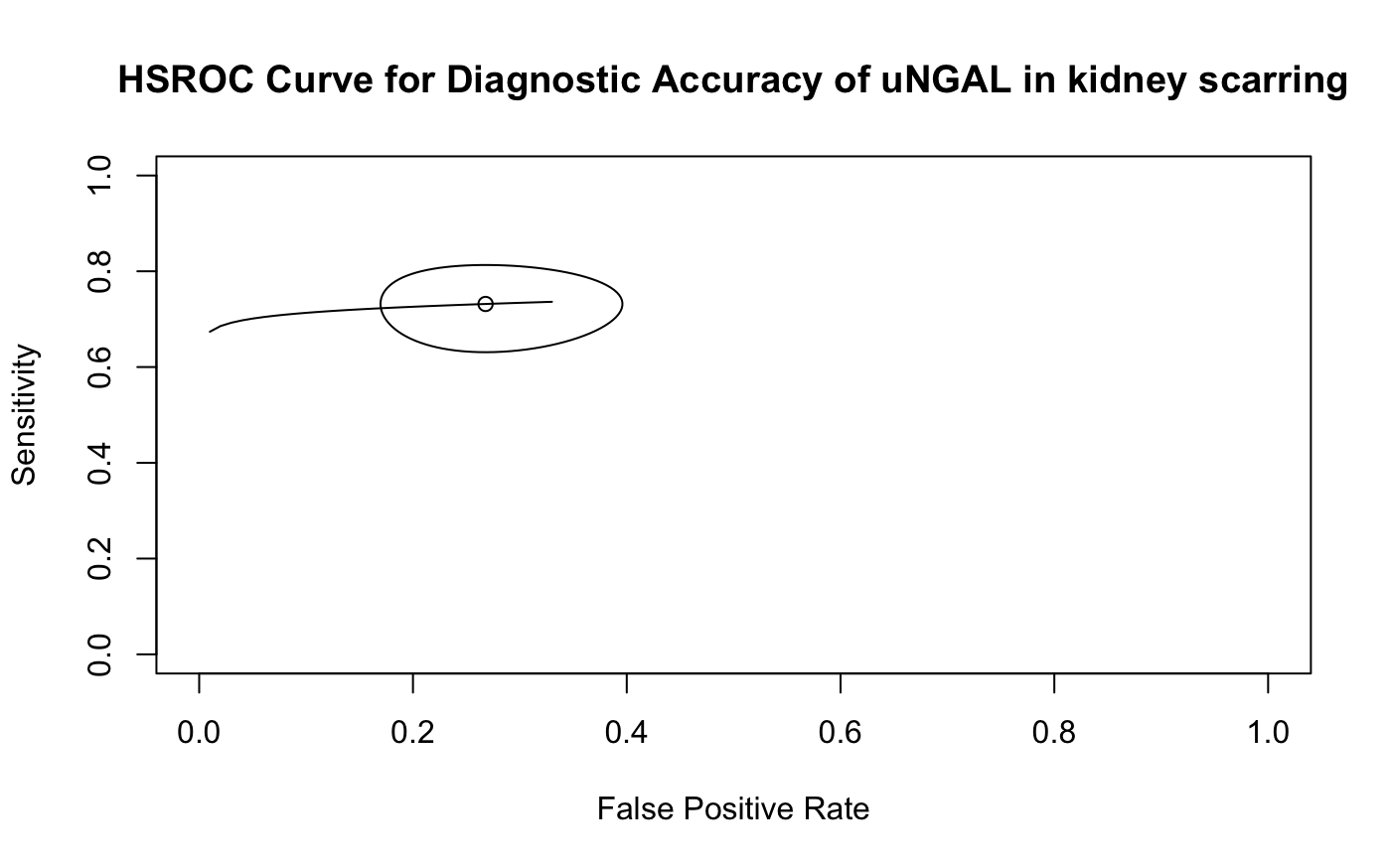

Supplement: Supplementary file 9 — ESM 9 (PNG 72.5 KB) [file 467_2025_6885_MOESM9_ESM.png]
