# Supplementary material for: The value of procalcitonin and urinary NGAL in the prediction of acute pyelonephritis and kidney scarring in pediatric patients with a history of febrile urinary tract infection: a systematic review and meta-analysis
Source: Pediatr Nephrol. 2025 Jul 31;41(2):323–37. doi: 10.1007/s00467-025-06885-0 (PMC12727856; doi:10.1007/s00467-025-06885-0)
Supplement: Supplementary file 10 — ESM 10 (DOCX 15.5 KB) [file 467_2025_6885_MOESM10_ESM.docx]

| **Number of records identified through searching electronic databases** |
| --- |
| **PubMed/MEDLINE** (02/01/2025) |
| (((((((((((((((pyelonephritis) OR (UTI)) OR (vesicoureteral reflux)) OR (VUR)) AND (neutrophil gelatinase associated lipocalin)) OR (NGAL)) OR (kidney injury molecule-1)) OR (KIM-1)) OR (liver fatty acid binding protein)) OR (LFABP)) OR (pentraxin 3)) OR (PTX3)) OR (procalcitonin)) AND (kidney scarring)) ) OR (renal scar) |
| **Results**: 1978 |
| **Scopus** (02/01/2025) |
| ( "pyelonephritis" ) OR ( " UTI" ) OR ( "vesicoureteral reflux" ) OR ( "VUR" ) AND ( "neutrophil gelatinase associated lipocalin" ) OR ( "NGAL" ) OR ( "kidney injury molecule-1" ) OR ( "KIM-1" ) OR ( "liver fatty acid binding protein" ) OR ( "LFABP" ) OR ( "pentraxin 3" ) OR ( "PTX3" ) OR ( "procalcitonin" ) AND ( "kidney scarring" ) OR ( "renal scar" ) |
| **Results:** 313 |
