# Supplementary material for: The value of procalcitonin and urinary NGAL in the prediction of acute pyelonephritis and kidney scarring in pediatric patients with a history of febrile urinary tract infection: a systematic review and meta-analysis
Source: Pediatr Nephrol. 2025 Jul 31;41(2):323–37. doi: 10.1007/s00467-025-06885-0 (PMC12727856; doi:10.1007/s00467-025-06885-0)
Supplement: Supplementary file 11 — ESM 11 (DOCX 17.6 KB) [file 467_2025_6885_MOESM11_ESM.docx]

**Appendix Table 2. Excluded studies**

| **Study** | **Reason for exclusion** |
| --- | --- |
| Baranton E, Ribet C, Freyssinet E, Bernardor J, Boyer C, Lavrut-Hollecker F, Demonchy D, Schuler E, Fontas E, Tran A. Plasma and Urinary Neutrophil Gelatinase-Associated Lipocalin as Predictors of Renal Parenchymal Involvement in Children with Febrile Urinary Tract Infection: A Pilot Study. Children (Basel). 2024 Sep 3;11(9):1081. doi: 10.3390/children11091081. PMID: 39334613; PMCID: PMC11429667. | Wrong outcome |
| Bigot S, Leblond P, Foucher C, Hue V, D'Herbomez M, Foulard M. Apport du dosage de la procalcitonine pour le diagnostic de pyélonéphrite aiguë de l'enfant [Usefulness of procalcitonin for the diagnosis of acute pyelonephritis in children]. Arch Pediatr. 2005 Jul;12(7):1075-80. French. doi: 10.1016/j.arcped.2005.03.058. PMID: 15893462. | Wrong language |
| Gavrilovici C, Dusa CP, Iliescu Halitchi C, Lupu VV, Spoiala EL, Bogos RA, Mocanu A, Gafencu M, Lupu A, Stoica C, Starcea IM. The Role of Urinary NGAL in the Management of Primary Vesicoureteral Reflux in Children. Int J Mol Sci. 2023 Apr 26;24(9):7904. doi: 10.3390/ijms24097904. PMID: 37175609; PMCID: PMC10177906. | Wrong study type |
| Kotoula A, Gardikis S, Tsalkidis A, Mantadakis E, Zissimopoulos A, Kambouri K, Deftereos S, Tripsianis G, Manolas K, Chatzimichael A, Vaos G. Procalcitonin for the early prediction of renal parenchymal involvement in children with UTI: preliminary results. Int Urol Nephrol. 2009;41(2):393-9. doi: 10.1007/s11255-008-9472-2. Epub 2008 Oct 3. PMID: 18836845. | Wrong outcome |
| Leroy S, Adamsbaum C, Marc E, Moulin F, Raymond J, Gendrel D, Bréart G, Chalumeau M. Procalcitonin as a predictor of vesicoureteral reflux in children with a first febrile urinary tract infection. Pediatrics. 2005 Jun;115(6):e706-9. doi: 10.1542/peds.2004-1631. Epub 2005 May 2. PMID: 15867014. | Wrong outcome |
| Yamanouchi S, Kimata T, Akagawa Y, Akagawa S, Kino J, Tsuji S, Kaneko K. Reduced urinary excretion of neutrophil gelatinase-associated lipocalin as a risk factor for recurrence of febrile urinary tract infection in children. Pediatr Nephrol. 2021 Jun;36(6):1473-1479. doi: 10.1007/s00467-020-04863-2. Epub 2021 Jan 7. PMID: 33411073. | Wrong outcome |
| Yun BA, Yang EM, Kim CJ. Plasma Neutrophil Gelatinase-Associated Lipocalin as a Predictor of Renal Parenchymal Involvement in Infants With Febrile Urinary Tract Infection: A Preliminary Study. Ann Lab Med. 2018 Sep;38(5):425-430. doi: 10.3343/alm.2018.38.5.425. PMID: 29797812; PMCID: PMC5973916. | Wrong outcome |
| Usefulness of procalsitonin and C-reactive protein rapid tests for the management of children with urinary tract infection. Pediatr Infect Dis J. 2001; 20.507-11 | Wrong outcome |
