# Supplementary material for: The value of procalcitonin and urinary NGAL in the prediction of acute pyelonephritis and kidney scarring in pediatric patients with a history of febrile urinary tract infection: a systematic review and meta-analysis
Source: Pediatr Nephrol. 2025 Jul 31;41(2):323–37. doi: 10.1007/s00467-025-06885-0 (PMC12727856; doi:10.1007/s00467-025-06885-0)
Supplement: Supplementary file 12 — ESM 12 (DOCX 22.5 KB) [file 467_2025_6885_MOESM12_ESM.docx]

| Study ID | STROBE score | % | Quality grade |
| --- | --- | --- | --- |
| Amiri 2022 | 17/22 | 77.3 | Good |
| Becerir 2019 | 15/22 | 68.2 | Fair |
| Belhadj-Tahar 2008 | 13/22 | 59.1 | Fair |
| Benador 1998 | 14/22 | 63.6 | Fair |
| Benzer 2017 | 18/22 | 81.8 | Good |
| Bressan 2009 | 17/22 | 77.3 | Good |
| Colceriu 2023 | 14/22 | 63.6 | Fair |
| Eskandarifan 2023 | 17/22 | 77.3 | Good |
| Gurgoze 2005 | 12/22 | 54.5 | Fair |
| Guven 2006 | 16/22 | 72.7 | Good |
| Han 2022 | 14/22 | 63.6 | Fair |
| Ichino 2010 | 15/22 | 68.2 | Fair |
| Karavanaki 2007 | 12/22 | 54.5 | Fair |
| Kitao 2015 | 15/22 | 68.2 | Fair |
| Kotoula 2009 | 16/22 | 72.7 | Good |
| Lee 2020 | 18/22 | 81.8 | Good |
| Naik 2022 | 18/22 | 81.8 | Good |
| Orive 2012 | 05/22 | 22.7 | Poor |
| Parmaksız 2016 | 16/22 | 72.7 | Good |
| Pecile 2004 | 11/22 | 50.0 | Fair |
| Penedo 2021 | 17/22 | 77.3 | Good |
| Prat 2003 | 12/22 | 54.5 | Fair |
| Rafiei 2015 | 14/22 | 63.6 | Fair |
| Sheu 2011 | 15/22 | 68.2 | Fair |
| Smolkin 2002 | 09/22 | 40.1 | Poor |
| Toker 2013 | 15/22 | 68.2 | Fair |
| Tuerlinchx 2005 | 10/22 | 45.4 | Poor |
| Yamanouchi 2018 | 16/22 | 72.7 | Good |
| Abbreviations: ID: identification; %: percentage | | | |
